# Effect of Combined Endurance Training and MitoQ on Cardiac Function and Serum Level of Antioxidants, NO, miR-126, and miR-27a in Hypertensive Individuals

**DOI:** 10.1155/2022/8720661

**Published:** 2022-01-13

**Authors:** Yaser Masoumi-Ardakani, Hamid Najafipour, Hamid Reza Nasri, Soheil Aminizadeh, Shirin Jafari, Daruosh Moflehi

**Affiliations:** ^1^Physiology Research Center, Institute of Neuropharmacology, Kerman University of Medical Sciences, Kerman, Iran; ^2^Cardiovascular Research Center, Institute of Basic and Clinical Physiology Sciences, Kerman University of Medical Sciences, Kerman, Iran; ^3^Endocrinology and Metabolism Research Center, Institute of Basic and Clinical Physiology Sciences, and Department of Physiology and Pharmacology, Afzalipour School of Medicine, Kerman University of Medical Sciences, Kerman, Iran; ^4^Department of Exercise Physiology, Faculty of Physical Education and Sport Sciences, Shahid Bahonar University of Kerman, Kerman, Iran

## Abstract

**Objectives:**

Hypertension (HTN) is one of the most important risk factors for cardiovascular diseases. Despite advances in treatment and control of HTN, the prevalence of HTN is still increasing. MitoQ is a supplement that acts on mitochondria and attenuates reactive oxygen species (ROS), which plays an important role in cardiovascular health. miRNAs play an important role in the pathophysiology of HTN. We evaluated the effects of MitoQ supplementation and endurance training (ET), alone and in combination, on functional indices of the heart and serum levels of miR-126, miR-27a, antioxidants, and NO, in patients with HTN.

**Methods:**

In a double-blind randomized clinical trial, 52 male participants (age 40-55 years) were randomly divided into four groups (*n* = 13) of placebo, MitoQ (20 mg/day, oral), ET (cycle ergometer, moderate intensity, 40-60% VO_2_ peak, heart rate 120-140 b/min, 45 min a day, three days/week for six weeks), and MitoQ+ET. Cardiac function indices were assessed by echocardiography before and after interventions.

**Results:**

Systolic blood pressure (SBP) significantly decreased in all intervention groups (*P* < 0.001) while DBP (*P* < 0.01) and LV hypertrophy (*P* < 0.05) were significantly decreased only in the MitoQ+ET group. Serum levels of SOD, GPx, and NO and the level of miR-126 significantly increased in all treatment groups, while miR-27a reduced in the ET (*P* < 0.05) and MitoQ+ET (*P* < 0.01) groups.

**Conclusions:**

Compared to MitoQ and ET alone, their combination has more prominent improving effects on cardiac health and amelioration of BP in the patients with HTN. These effects are through miR-126 and miR-27a modulation and ameliorating mitochondrial ROS production.

## 1. Introduction

Hypertension (HTN) is one of the most important risk factors for coronary artery disease (CAD) and one of the leading causes of mortality worldwide. About 45% of deaths due to cardiac disease and 51% of deaths due to stroke are related to HTN [1]. Despite medical advances in recent decades in treating HTN, 80 million adults in the United States suffer from HTN [2], and the prevalence of HTN is still rising in many countries. From half of those who receive treatment against HTN, the disease is uncontrolled [3]. The prevalence is related to demographic, genetic, and lifestyle especially the level of physical activity [4].

Regular and continuous aerobic exercise can control blood pressure (BP) and prevent progress to HTN in prehypertensive individuals [5]. Long-term or endurance training (ET) results in morphological and physiological adaptations in the cardiovascular system, such as increase in aerobic capacity and contractile strength and reduction in heart rate [5]. In the spontaneously hypertensive rats, exercise reduced systolic blood pressure and increased eNOS expression and nitric oxide (NO) production [6]. Beneficial effects of exercise have also been shown to be through altering the expression of microRNAs (miRNAs) and genes of the renin-angiotensin-aldosterone system (RAAS) and mitochondrial biogenesis [7–9]. Reactive oxygen specious (ROS) and other oxidants have a pivotal role in pathophysiology of CVDs including abnormalities in vessel structure and function, leading to HTN [10]. ET has antioxidant properties and improves endothelial function. Relatedly, mitoquinone mesylate (MitoQ) is a mitochondrial-targeted antioxidant supplement that eliminates ROS and improves vascular function [11]. MitoQ supplementation for 4 weeks in mice has restored age-related increase in aortic stiffness [12] and reduced angiotensin 2 level [13]. However, the effect of MitoQ has not been investigated on the blood pressure of patients with HTN yet.

MicroRNAs have been identified as important regulators of gene expression [14], e.g., the enzymes involved in generation and scavenging of ROS, in production of nitric oxide synthase (NOS), SOD, and GPx [15]. miRNAs play an important role in the pathophysiology of HTN and are increasingly being introduced as new biomarkers in CVD [16]. It was reported that serum levels of miR-9 and miR-126 are lower in patients with HTN [17], and the improving effects of exercise on HTN are induced by altering the expression of miRNAs [7, 8]. In this regard, miR-126 has a strong association with RAAS [7, 8], and miR-27a interferes with the control of BP by affecting angiotensin-converting enzyme (ACE) gene [18]. On the other hand, miR-126 is one of the most abundant miRNAs in endothelial cells [19], associating with the level of blood pressure [20], in which its deletion causes damage to vascular integrity [21]. Fernandes and colleagues showed a lower level of miR-126 in spontaneously hypertensive rats, which was recovered by exercise [22]. Therefore, it seems that there is an interrelationship among oxidative stress (ROS production), oxidant/antioxidant status, and miRNAs in many diseases including HTN [15, 23].

Considering the central role of mitochondria in ROS production, the important role of ROS in HTN, and the pivotal role of miRNAs in regulation of oxidant/antioxidant system and pathophysiology of HTN, this clinical trial is aimed at assessing whether the mitochondrial-targeted antioxidant MitoQ, alone or in combination with aerobic ET, has beneficial effects in improving the blood pressure of HTN patients. We also assessed cardiac function, biochemical factors, the level of NO and antioxidants, and the serum level of miR-126 and miR-27a of the patients with HTN as probable mechanistic tools.

## 2. Material and Methods

### 2.1. Materials

The material used and their sources were as follows: MitoQ capsule (MitoQ Ltd, New Zealand), superoxide dismutase (SOD) kit (Randox, #RS504, UK), glutathione peroxidase (GPx) kit (Randox, #SD125, UK), nitric oxide (NO), RNA isolation kit (Norgen Biotek, #17200, Canada), cDNA synthesis kit (Norgen Biotek, #54410, Canada), cel-miR-39 (Norgen Biotek, #59000, Canada), SYBR green (Ampliqon, #A325402, Denmark), universal primer (reverse) (Norgen Biotek, #59000, Canada), and primers (MetaBion, Germany).

### 2.2. Subjects

In this double-blind randomized clinical trial, 52 male middle-aged HTN patients (40-55 years old) were selected from individuals who participated in the KERCADRS (Kerman Coronary Artery Disease Risk Factor Study) or patients referred to the Cardiovascular Department of Shafa Hospital, Kerman, Iran. We selected male subjects because in this age range, many women experience menopause that affects the level of their sex hormones, and this may affect the results of the study as a confounding factor. All procedures, goals of the study, and the potential benefits and risks of ET or MitoQ were described to the participants, and an informed consent form was signed by them. The study protocol was according to the standards set by the latest revision of the Declaration of Helsinki and was verified by the ethics committee of Kerman University of Medical Sciences (IR.KMU.REC.1397.595 and IRCT20190228042870N1).

The criteria for HTN were according to the guidelines of the European Heart Association as follows: SBP ≥ 140 mmHg and/or DBP ≥ 90 mmHg. We entered the patients with moderate HTN to the study as ET may be a cardiovascular risk for subjects with severe HTN. Other exclusion criteria were liver, kidney, and lung diseases, diabetes, obesity (BMI ≥ 30 kg/m^2^), cancer, and known cardiovascular diseases other than HTN (such as valvular heart diseases and heart failure). The patient's demographic information including age, sex, medical history, family history of HTN, physical activity (including type of activity, frequency, and duration), alcohol consumption, and using any medication were collected using a validated questionnaire being used in KERCADRS. Cardiac function was assessed by two-dimensional mode echocardiography (Philips Ultrasound, EPIQ, USA).

The participants were randomly divided into 4 groups (*n* = 13): placebo, MitoQ (20 mg/day orally, once a day) [11], endurance training (ET), and MitoQ+ET. The groups were matched according to BMI, sex, and age. ET (moderate intensity, 40 to 60% VO_2_ peak, heart rate 120-140 b/min, duration 45 min) was performed for 6 weeks (3 sessions per week). The blood samples were taken after an overnight fasting, before (on day 1) and at the end of the study (day 43). The samples were centrifuged at 5000 rpm for 10 minutes, and the serum was aliquoted and stored at −80°C for future quantifications. A part of the serum was used for biochemical tests: FBS, creatinine, albumin, SGPT, SGOT, alkaline phosphatase (ALP), triglyceride (TG), high-density lipoprotein cholesterol (HDL-C), and total cholesterol (TC). Low-density lipoprotein cholesterol (LDL-C) level was calculated by the Friedewald formula: LDL − C = TC–[HDL − C − (TG/5)].

Another part of the serum was used to determine miRNAs and also SOD, GPx, and NO by the ELISA method.

### 2.3. Blood Pressure Measurement

Baseline BP was measured twice (30 min apart) in an upright sitting position, after at least 10 minutes at rest, and the values were averaged. All subjects were asked to avoid consuming caffeinated beverages such as coffee, tea, soft drinks, supplements, and alcohol at least two hours before BP measurement. The recordings were made under quiet and comfortable ambient (temperature ~24°C) conditions with an automated device (Omron, M6 Comfort, Japan) to avoid the possibility of investigator bias in measurements.

### 2.4. Body Composition Measurements

Body weight was measured using a medical beam balance (Allegro Medical, USA), and body mass index (BMI) was calculated as weight (kg) divided by height squared (m^2^) and used to differentiate between normal weight (BMI < 25) and overweight (BMI ≥ 25 − 29.9). For assessing body fat, we used a caliper (Saehan Skinfold Caliper, South Korea) for measuring skinfolds thickness in seven points. The Jackson and Pollock equation formula was used for calculating the percent of body fat as follows [24].

(1) $$\begin{array}{c} \text{Body fat }\left(\%\right)=495/\left(1.112-\left(0.00043499s\right)+\left(0.00000055ss\right)-\left(0.00028826a\right)\right)-450. \end{array}$$

where *s* is the sum of skinfolds (seven points) and *a* is age.

### 2.5. Modified Astrand-Rhyming Cycle Ergometer Test

**Figure 1:**
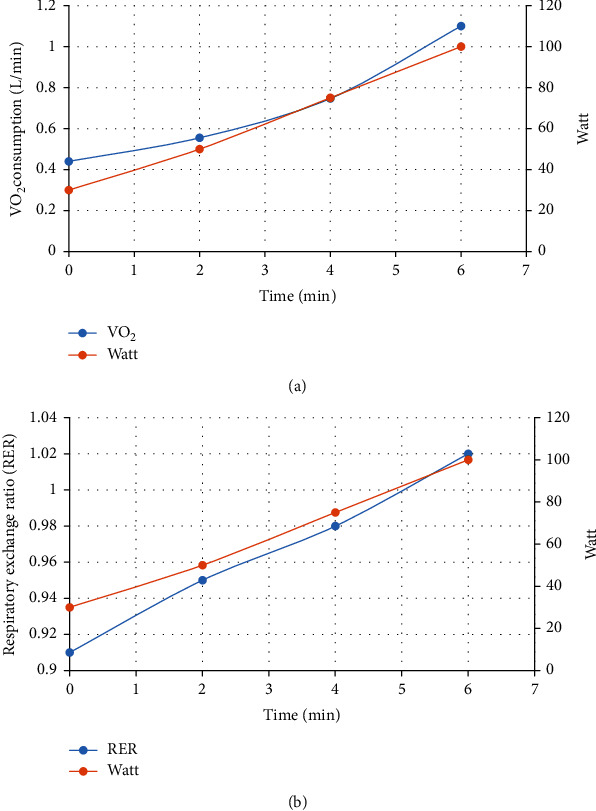
Oxygen uptake kinetic (a) and RER changes (b) in groups with endurance training (Astrand test).

Before ET protocol, all subjects performed a cardiopulmonary exercise test (CPET) for determining the peak power and VO_2_ peak by the Astrand test. Subjects were also asked to avoid the ingestion of alcohol, cigarette, and caffeine-containing products and to refrain from strenuous activity for at least 12 h before the test. The Astrand test was conducted on an upright cycle ergometer (Monark, Ergomedic 839 E, Sweden) coupled to a gas analyzer (Cortex, METALYZER 3B, Germany). The Astrand test consisted of a steady-state resting period, then 2 min of warm-up without load, followed by a constant protocol of six minutes in length. During this time, he attempted to maintain his heart rate between 120 and 170 bpm. The pedal rate was 50 ± 5 rpm for the duration of the 6 min [25]. Subjects were asked to score their sense of breathlessness and muscle fatigue throughout the exercise and at its peak, using the Borg scale [26]. Oxygen saturation (SpO_2_) by pulse oximeter (Beurer, Germany), electrocardiographic monitoring of heart rate (HR), and BP, oxygen uptake (VO_2_), carbon dioxide production (VCO_2_), respiratory exchange ratio (RER), and ventilation (V′E) were recorded. The average of HRs and the final Watts were used to predict VO_2_ peak from a nomogram, and an age correction factor was applied [27]. Successful tests were defined as a participant had completed the 6 min test at a workload to induce HRs within the range of 120–140 bpm (Figure 1).

### 2.6. Endurance Training Protocol (moderate intensity)

ET was applied in training groups for 6 weeks (3 sessions per week). Based on the output Watt in the Astrand test, the first training session lasted 15 minutes for each subject (at 40 to 60% maximum Watt output). In the next sessions, the time and intensity of exercise training were gradually increased till the time period reached about 45 minutes at the 12th session (the end of the fourth week), and during this period, the heart rate was between 120 and 140 beats/min. The intensity of exercise training was maintained constant at duration of 45 minutes in the fifth and sixth weeks [28].

SBP, DBP, arterial SpO_2_, and HR were taken from participants before and during exercise (peak) and at the end of recovery period.

### 2.7. Cardiac Function Assessment

Cardiac function parameters, left ventricle ejection fraction (EF), left ventricle shortening fraction (SF), and left ventricular hypertrophy (LVH), were obtained by guided M-mode frames. The parameters were assessed at baseline and the day after the end of the study.

In accordance with the American Society of Echocardiography guidelines [29], we used the greater of two measurements recorded from the interventricular septum and posterior wall thickness obtained by echocardiography to classify the hypertensive patients as having no LVH (<1.1 cm), mild LVH (1.1–1.3 cm), moderate LVH (1.4–1.6 cm), and severe LVH (≥1.7 cm).

### 2.8. Determination of SOD Activity

SOD activity in the serum was determined by the Randox kit based on the manufacturers' instruction. SOD functions as a catalyst in the dismutation of O_2_ radicals into hydrogen peroxide (H2O2) and converting NBT to NBT-diformazan that absorbs light at 560 nm [30].

### 2.9. Determination of GPx Activity

GPx activity was determined by the Randox kit according to the method described by Paglia and Valentine. The assay kit measures GPx activity indirectly by a coupled reaction with glutathione reductase, the enzyme responsible for regenerating the reduced form of oxidized glutathione (GSSG). Absorbance decreases at 340 nm when NADPH is oxidized to NADP^+^ [31].

### 2.10. Determination of NO Level

The Griess method was used to measure the level of NO in serum. Serum deproteinization was performed initially using ZnSO4 in the presence of 0.3 M NaOH. Then Vanadium three chloride (VaCl_3_) (that converts nitrate into nitrite) and the Griess reagent were mixed with deproteinated serum and incubated at 37°C for 30 min. Finally, the optical density (OD) was measured at 540 nm [32].

### 2.11. miR-126 and miR-27a Measurement by RT-qPCR

Total RNA was isolated from the serum using the total RNA purification kit. Briefly, RNA was isolated from 150 *μ*l of serum using the RL buffer washed and eluted in RNAse free water. RNA concentration and purity were quantified using NanoDrop ND-2100 (Thermo Fisher Scientific, USA). To normalize between samples, 3.5 *μ*l *Caenorhabditis elegans* miR-39 (cel-miR-39) was added to each sample. Immediately after RNA isolation, 5 *μ*l of RNA was reverse transcribed using the microScript microRNA cDNA synthesis kit. cDNA was PCR-amplified (StepOnePlus instrument, Applied Biosystems, USA) using RealQ Plus Master Mix Green, high ROX and miRNA-specific primers for miR-126 and miR-27a. All samples were assayed in duplicate. Relative expression level for a given miRNA was normalized to cel-miR-39 as external control. The expression was calculated as fold change according to the formula: fold change = 2^–ΔΔCT^ in which ΔΔCT = [(CT gene − CT cel − miR − 39)_treatment_–[CT gene − CT cel − miR − 39)]_CTL_ [33]. The forward primer sequences of miRs were as follows: miR-126: 5′-TCGTACCGTGAGUTATAATGCG-3′, miR-27a: 5′-TTCACAGTGGCTAAGTTCCGC-3′, and cel-miR-39: 5′-UCACCGGGUGUAAAUCAGCUUG-3′.

We used a universal primer that was supplied by the company as reverse in the reactions.

### 2.12. Statistical Analysis

The data were analyzed by the SPSS software (SPSS version 26, SPSS Inc., Chicago, IL, USA) and GraphPad Prism (GraphPad v.8.4.3., San Diego, LLC, USA). First, the data distribution was examined by the Kolmogorov-Smirnov test and if it was normal. Two-way repeated measures ANOVA was used to compare BP, antioxidants, and NO level, followed by Tukey's post hoc test for between-group comparisons. Nonparametric equivalent tests were used when the distribution of the data was not normal. Comparison of baseline ventilation indices (VO_2_ Peak, VE/VO_2_, and VE/VCO_2_) between two ET groups was performed by unpaired *t*-test. Chi-square test was used for descriptive statistics (history of HTN, smoking, alcohol consumption, and physical activity). A *p* < 0.05 was considered significance level.

## 3. Results

### 3.1. Anthropometric, Demographic, and General Characteristics of the Study Population

Table 1 shows that the study groups were similar in demographic and general characteristics at baseline. Regarding the body weight (in kg), the ET (81 ± 2.4 vs. 80 ± 2.0) and MitoQ+ET (83 ± 1.7 vs. 81 ± 1.7) groups experienced a significant reduction of weight (*p* < 0.05) during the study period compared to their baseline (Table 1).

### 3.2. Clinical, Ventilatory, and Body Fat Variables

Resting HR was 76 ± 1.8, 76 ± 1.9, 72 ± 1.8, and 74 ± 2.0 b/min in placebo, MitoQ, ET, and MitoQ+ET groups, respectively. HR at peak was 127 ± 2.5 in ET and 132 ± 2.5 in MitoQ+ET groups at the follow-up. Arterial SpO_2_ was 95 ± 0.1, 93 ± 0.2, 95 ± 0.2, and 95 ± 0.2 percent in placebo, MitoQ, ET, and MitoQ+ET groups, respectively, at the baseline and 94 ± 0.2 in ET and 93 ± 0.2 in MitoQ+ET groups at the follow-up. Also, ventilation and lung function indices of VO_2_ Peak, VE/VO_2_, VE/VCO_2_, and RER showed no significant difference among the groups at the onset of the study (Table 2). Abdominal and supraspinal fat in MitoQ, ET, and MitoQ+ET groups significantly reduced. In the case of body fat percentage, only ET and MitoQ+ET groups showed a significant decrease during fallow-up (*p* < 0.05 and *p* < 0.05) compared to their baseline (Table 2).

### 3.3. Measurement of Serum Biochemical Variables

Serum TG levels significantly reduced (*p* < 0.05) only in the combined group of MitoQ+ET. In MitoQ, ET, and MitoQ+ET groups, serum cholesterol and creatinine levels decreased significantly compared to their baselines (Table 3).

### 3.4. Effects of MitoQ and ET on SBP and DBP

**Figure 2:**
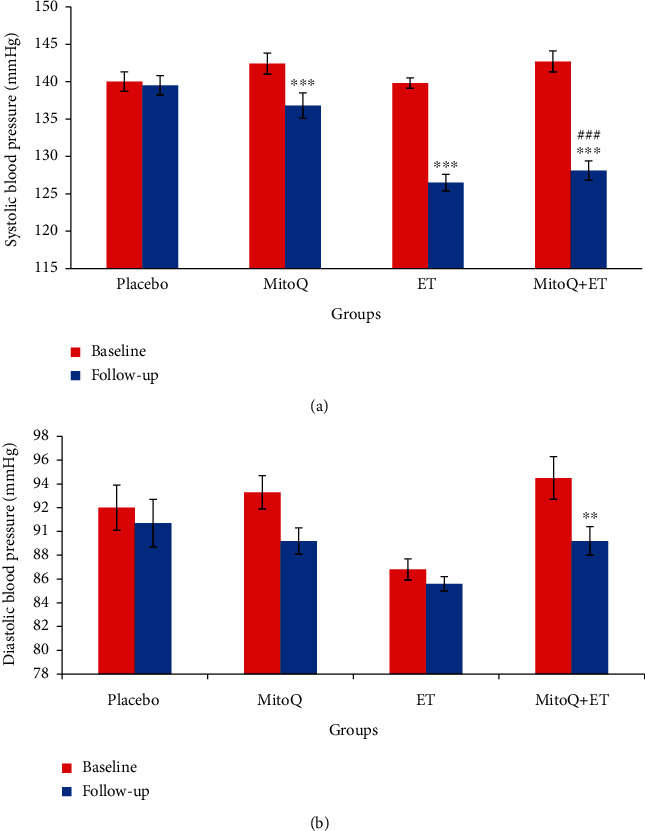
Baseline and follow-up systolic and diastolic blood pressure values (a, b) in the four groups of the study after 6 weeks of ET and MitoQ+ET supplementation (*n* = 13 in each group). Data are expressed as mean ± SEM. ^∗∗^*p* < 0.01, ^∗∗∗^*p* < 0.001 vs. related baseline. #Significant difference vs. MitoQ follow-up. ET: endurance training.

Based on the data presented in Figure 2, in MitoQ, ET, and MitoQ+ET groups, SBP decreased significantly compared to its baseline (*p* < 0.001) (Figure 2(a)). However, DBP only in the MitoQ+ET group showed a significant decrease (*p* < 0.01) (Figure 2(b)).

### 3.5. Effects of Interventions on Left Ventricular Systolic Function

**Figure 3:**
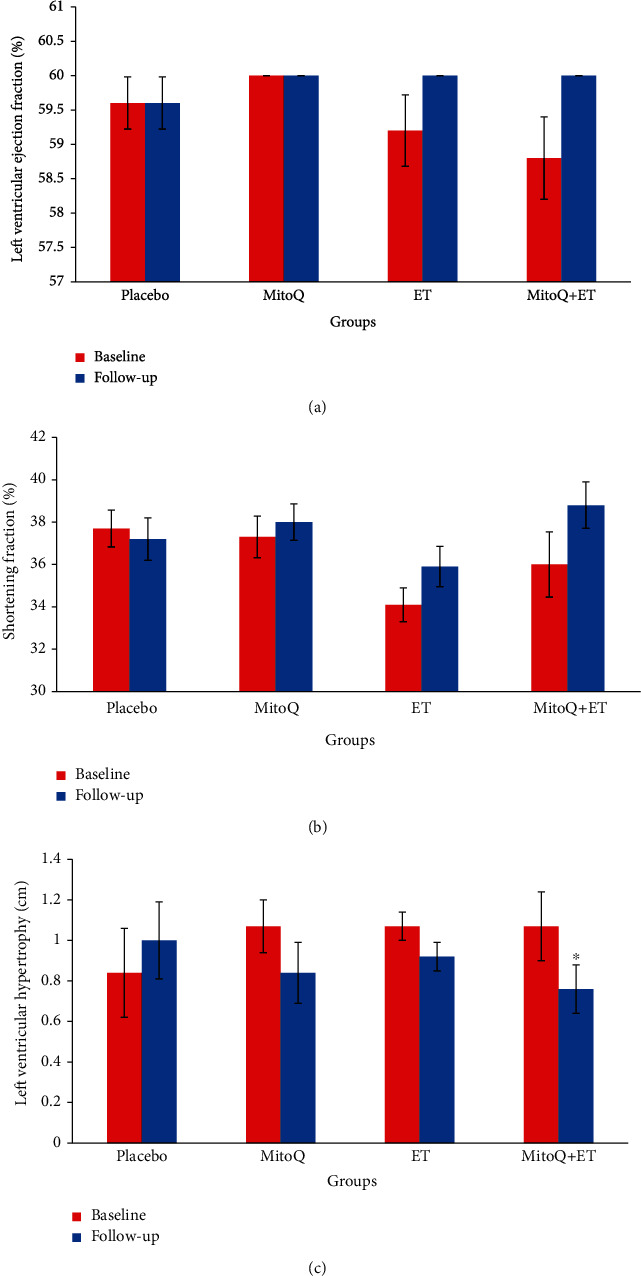
Cardiac function parameters at baseline and follow-up in 6 weeks of ET and MitoQ+ET supplementation (*n* = 13 in each group). Data are expressed as mean ± SEM: (a) left ventricular ejection fraction, (b) shortening fraction (%), and (c) left ventricular hypertrophy. ^∗^Significant difference vs. corresponding baseline. ET: endurance training.

Figure 3 shows the left ventricular systolic function in different studied groups. The ejection fraction and shortening fraction did not show significant changes (Figures 3(a) and 3(b)), but the LVH in the combined group (MitoQ+ET) significantly decreased (Figure 3(c)).

### 3.6. Effects of Interventions on Antioxidant Enzymes and NO

**Figure 4:**
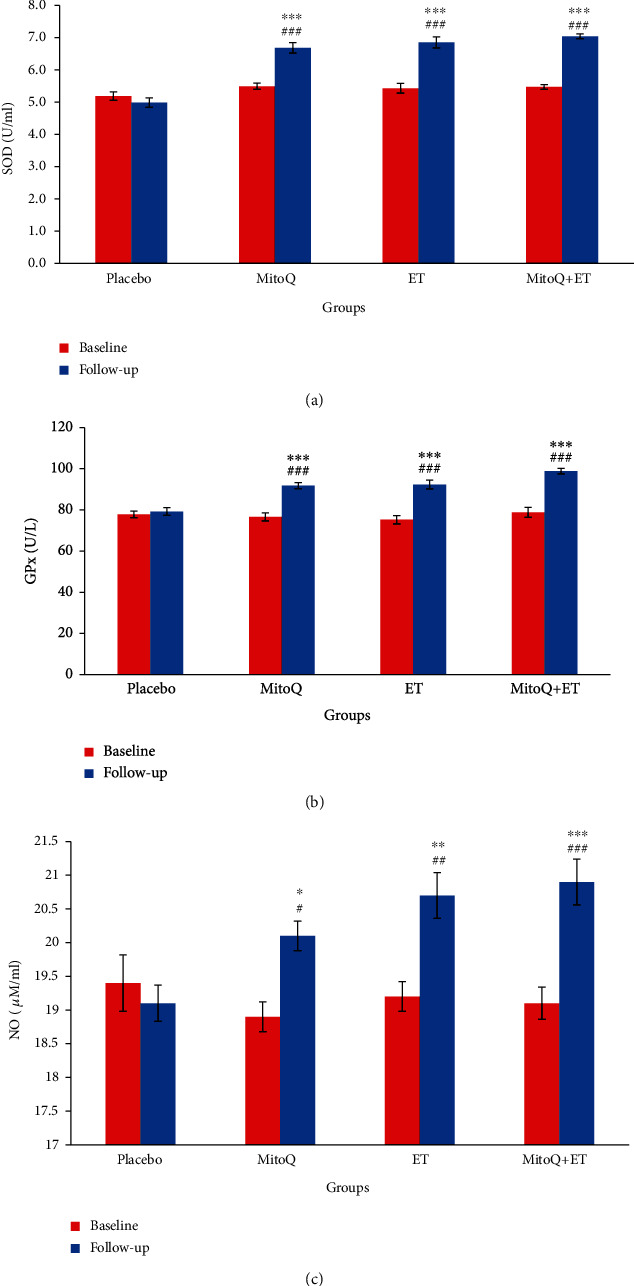
Serum antioxidant enzymes (a, b) and NO (c) levels at baseline and follow-up of 6 weeks ET and MitoQ+ET supplementation in patients with HTN. Values are presented as mean ± SEM. *n* = 13 in each group. ^∗^*p* < 0.05, ^∗∗^*p* < 0.01, ^∗∗∗^*p* < 0.001 vs. related baseline. ^#^*p* < 0.05, ^##^*p* < 0.01, ^###^*p* < 0.001 vs. the placebo group. ET: endurance training.

After 6 weeks, serum SOD and GPx activity increased in all three intervention groups compared to the placebo group and also compared to their related baseline value (*p* < 0.001 in all cases) (Figures 4(a) and 4(b)). There was no difference among the baseline value of these antioxidants in all groups. MitoQ, ET, and combination of MitoQ+ET caused a significant increase in serum NO level compared with their baseline values and compared to the placebo group (Figure 4(c)). MitoQ+ET had more prominent effect on NO level than ET or MitoQ alone.

### 3.7. Effects of MitoQ and ET on Serum miR-126 and miR-27a

**Figure 5:**
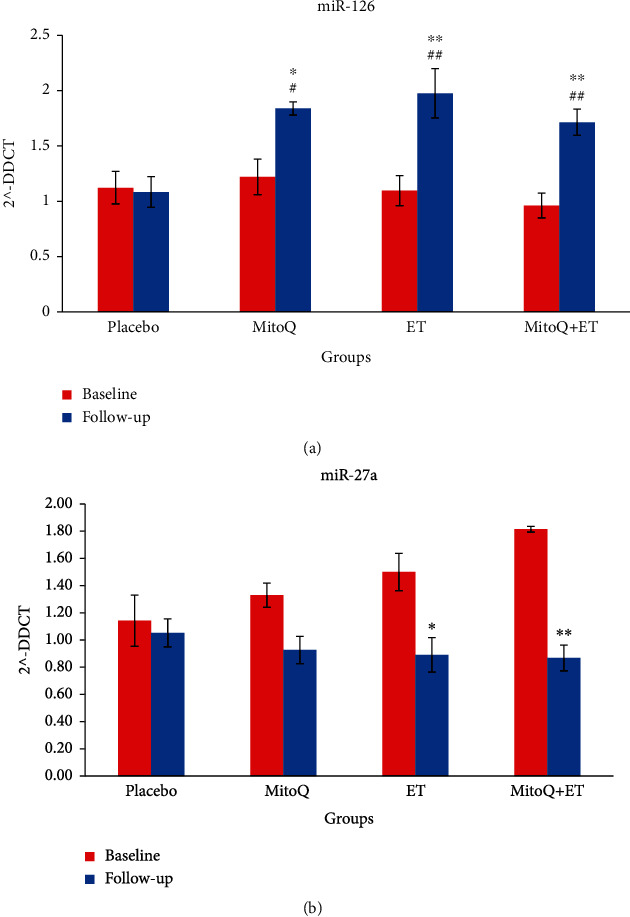
Relative expression (mean ± SEM) of miR-126 and miR-27a in the serum of patients with hypertension, and the effect of treatment with MitoQ, ET, and MitoQ+ET after 6 weeks follow-up. *n* = 13 in each group. ^∗^*p* < 0.05, ^∗∗^*p* < 0.01 vs. related baseline values, ^#^*p* < 0.05, ^##^*p* < 0.01 vs. the placebo group. ET: endurance training.

Interventions increased miR-126 in MitoQ, ET, and MitoQ+ET combination groups and decreased miR-27a in ET and MitoQ+ET groups (Figures 5(a) and 5(b)). All interventions caused a significant increase in miR-126 compared with the placebo group (Figure 5(a)).

## 4. Discussion

In this study, the effects of 6 weeks ET or oral supplementation of MitoQ alone and their combination in hypertensive subjects were evaluated. The main finding was that combination therapy significantly reduced SBP and increased serum levels of SOD, GPx, and NO. The level of miR-126 significantly increased while the level of miR-27a significantly reduced in the combination group.

The results showed that serum levels of TG, TC, and LDL-C in the MitoQ+ET group significantly reduced and HDL-C significantly increased compared to baseline. It has been shown that exercise increases HDL-C and decreases LDL-C [34, 35], but administration of 20 mg of MitoQ for 6 weeks in adults had no effect on glucose, TG, TC, and HDL-C [11]. Here, MitoQ and ET alone had no significant effect on lipid profile except cholesterol. However, they potentiated the lipid lowering effect of each other when applied in combination (see Table 3). These findings show the beneficial effect combination therapy on lipid profile as a cardiovascular health variable. Also, there is no increase in kidney and liver function tests showing nonharmful effect of MitoQ on the body.

Regarding the effect of interventions on BP, the results showed that MitoQ+ET decreased SBP more than that of MitoQ alone. However, here, MitoQ has no additive effect with ET on reduction of BP when used in combination form. The reason may be that we entered the patients with moderate HTN to the study, and there was no possibility for additional effect of the two interventions because the baroreceptor reflex control system does not allow BP to come below normal levels. Probably in more severe hypertensive subjects, combination therapy may show more beneficial effects.

There is evidence that reduced antioxidant defense system and increasing oxidative stress contribute to HTN development. Mitochondria are one of the main sites for the generation of free radicals due to their oxidative phosphorylation process [36], and overproduction of superoxide radicals in the mitochondria and decreased SOD enzyme activity lead to HTN. In people with HTN, increased oxidative stress activates RAAS, which in turn reduces the bioavailability of NO in renal microvasculature [4]. We found that 6 weeks of ET increased NO, SOD, and GPx in the serum of individuals in all three groups. Aerobic exercise increases shear stress and therefore increases NO production in vascular endothelium and improves vascular structure [37]. Also, exercise has an effect on cardiac metabolic remodeling through eNOS, especially as it increases mitochondrial biogenesis [9], and prevent lipid peroxidation and mitochondrial damage [38]. Exercise increases the expression of endothelin receptor type B (ETB) in the vascular endothelium, which increases vasodilation by increasing NO [39]. According to our results, ET caused significant increase in SOD, GPx, and NO levels, and its combination with MitoQ has added to these beneficiary effects. It seems that improvement in the redox status is a main reason for the significant decrease in SBP of our HTN patients. In this regard, MitoQ that is a mitochondria-targeted antioxidant may have exerted its protective effect against oxidative damage leading to ameliorating HTN [40]. Administration of 20 mg of MitoQ for 6 weeks has been found to be beneficial in lowering LDL-ox and improving aging-related cardiac mitochondrial dysfunction [11].

Our results showed that MitoQ significantly increased serum miR-126 levels, and ET did the same. miR-126 is critical for maintaining vascular homeostasis and integrity targeting the PI3K/AKT/eNOS pathway [41]. Decrease in its expression increases inflammation and endothelial dysfunction and decreases vascular repair capacity [33]. Circulating miR-126 levels have been found to be 55% lower in HTN patients compared to normotensive individuals [8, 33]. Fernandes et al. showed that miR-126 increased after exercise in hypertensive rats [22]. Based on the above researches, it may be postulated that the second mechanism that both MitoQ and ET reduced BP is probably increase in the expression of miR-126. In this regard, either of interventions has induced the full effect on miR-126 expression (Figure 5). Therefore, their combination had no extra effect on the level of this miRNA or on reduction of BP. miR-126 has been reported to induce cell proliferation and angiogenesis in nontumorigenic cells [42]. Although in this study MitoQ, ET, and their combination increased the miR-126 serum level compared to the placebo, this increase may have compensated its reduced level in our HTN patients [8, 33]. The fact that we did not find any increase in kidney and liver function tests assures us that the interventions planned in this study may have not harmful side effects on hypertensive subjects.

In the present study, we found that the miR-27a levels significantly reduced after performing ET, and in response to combination of ET with MitoQ. The reduction by MitoQ alone did not reach significant level. miR-27a has been shown to be reduced in circulation during chronic exercise [8]. ACE that is associated with HTN and causes left ventricular remodeling [8] is one of the targets of miR-27a. miR-27a increases ACE expression and thus leads to HTN by activating the NF*κ*B pathway causing cardiovascular inflammation and remodeling [43], ROS generation, and endothelial dysfunction [44]. miR-27a is a hypertrophic microRNA in the heart; and exercise has been shown to decrease miR-27a levels after 7 days [45]. The significant effect of MitoQ on miR-126 but its nonsignificant effect on miR-27a level may infer that unlike ET that induces its affects through both miR-126 and miR-27a, MitoQ mostly exerts its beneficial effects thorough miR-126. miR-27a has been proposed to be considered a cancer treatment target. As ROS production and reduction in antioxidant capacity are among proposed mechanisms in cancer production as well, it is possible that MitoQ plus ET is used for cancer treatment due to their antioxidant properties and reduction in the miR-27a level. We recommend to plan more studies about this property of MitoQ.

It was previously described that ET causes weight loss [8]. Our results showed that 6 weeks of ET caused weight reduction as well. MitoQ did not affect body weight and fat. Consistent with this finding, the study of Shill et al. showed that administration of 10 mg of MitoQ had no effect on body fat in young individuals [46]. Therefore, it does not seem that weight loss is a mechanism for reduction of BP by MitoQ.

## 5. Conclusion

Overall, considering the improvement of cardiovascular indices after six weeks of ET and oral MitoQ supplementation and considering that SOD, GPx, and NO increased and lipid profile and body fat was improved, it can be concluded that MitoQ supplementation in combination with this type and intensity of exercise may have positive effects on cardiovascular health indices in HTN patients. miR-126 and miR-27a can be considered potential targets of MitoQ in its beneficial effects on HTN and other cardiovascular risk factors.

## Figures and Tables

**Table 1:** Anthropometric, demographic, and general characteristics of the study population at baseline.

Variable	Placebo (*n* = 13)	MitoQ (*n* = 13)	ET (*n* = 13)	MitoQ+ET (*n* = 13)	*p* value
Age (years)	49 ± 0.7	49 ± 0.7	48 ± 0.9	47 ± 1.1	ns
Weight (kg)	76 ± 1.5	79 ± 2.0	81 ± 2.4	83 ± 1.7	ns
BMI (kg/m^2^)	26 ± 0.5	27 ± 0.5	26 ± 0.4	27 ± 0.4	ns
Family history in HTN, *N* (%)	12 (92.3)	13 (100)	13 (100)	13 (100)	ns
Current smoker, *N* (%)	6 (46.2)	3 (23.1)	2 (15.4)	2 (15.4)	ns
Physical activity, *N* (%)	8 (61.5)	9 (69.2)	5 (38.5)	4 (30.8)	ns
Ethanol intake (%)	0	0	0	0	ns

BMI: body mass index, HTN: hypertension, ET: endurance training. The data for age, weight, and BMI are presented as mean ± SEM.

**Table 2:** Baseline and follow-up (6 weeks) clinical, ventilatory, and body fat variables in hypertensive patients (*N* = 52).

Variable	Placebo		MitoQ		ET		MitoQ+ET	
	Baseline	Follow-up	Baseline	Follow-up	Baseline	Follow-up	Baseline	Follow-up
VO_2_ peak (ml/min)	NA	NA	NA	NA	3120 ± 138	NA	3030 ± 139	NA
VE/VO_2_	NA	NA	NA	NA	30.5 ± 0.83	NA	29.7 ± 0.83	NA
VE/VCO_2_	NA	NA	NA	NA	29.8 ± 0.56	NA	29.1 ± 0.57	NA
RER	NA	NA	NA	NA	1.02 ± 0.02	NA	1.01 ± 0.02	NA
Abdominal fat (mm)	32.5 ± 1.0	32.8 ± 1.2	33.2 ± 1.5	30.6 ± 1.2^∗^	37.5 ± 2.4	34.8 ± 2.2^∗^	39.8 ± 1.3	36.2 ± 1.3^∗∗∗###^
Supraspinal fat (mm)	27.7 ± 1.0	27.8 ± 1.2	29.0 ± 1.2	27.2 ± 1.4^∗^	27.9 ± 1.4	25.5 ± 1.6^∗∗∗^	32.9 ± 2	30.3 ± 1.7^∗∗∗###*δδδ*^
Breast fat (mm)	22.8 ± 0.8	22.9 ± 1.1	24.7 ± 0.8	24.2 ± 0.5	27.4 ± 0.8	26.2 ± 0.9	27.4 ± 0.8	27 ± 0.8^###^
Triceps fat (mm)	20.6 ± 0.7	20.8 ± 0.7	20.6 ± 0.7	19.4 ± 0.8	24.2 ± 0.6	23.3 ± 0.6	24.3 ± 0.9	23.3 ± 1.1^###^
Subscapular fat (mm)	23.4 ± 0.7	23.5 ± 0.8	22.2 ± 0.8	23.1 ± 0.8	24.5 ± 0.8	23.4 ± 0.7	25.3 ± 1.0	24.8 ± 1.1^##*δ*^
Calf fat (mm)	4.8 ± 0.7	4.7 ± 0.6	5.0 ± 0.5	4.8 ± 0.5	6.5 ± 0.8	5.9 ± 0.7	7.3 ± 0.7	6.6 ± 0.5^###^
Thigh fat (mm)	13.8 ± 0.28	13.9 ± 0.26	14.1 ± 0.45	13.8 ± 0.39	14.5 ± 0.47	14.2 ± 0.40	15.5 ± 0.43	15.4 ± 0.48
Body fat (%)	23.3 ± 0.7	23.4 ± 0.8	23.9 ± 0.8	23.2 ± 0.6	25.1 ± 0.7	23.7 ± 0.7^∗^	26.4 ± 0.8	24.9 ± 0.7^∗^

The data are expressed as mean ± SEM. ^∗^Significantly vs. baseline, ^#^significantly vs. MitoQ follow-up, *^*δ*^*significantly vs. ET follow-up. ET: endurance training, HR: heart rate, RER: respiratory exchange ratio, NA: not applicable.

**Table 3:** Baseline and follow-up (6 weeks) biochemical variables in hypertensive patients.

Variable	Placebo (*n* = 13)		MitoQ (*n* = 13)		ET (*n* = 13)		MitoQ+ET (*n* = 13)	
	Baseline	Follow-up	Baseline	Follow-up	Baseline	Follow-up	Baseline	Follow-up
FBS (mg/dl)	95.9 ± 1.4	96.3 ± 1.1	96.9 ± 1.1	96.8 ± 1.0	97.7 ± 1.4	94.2 ± 0.9	94.8 ± 1.7	92.7 ± 1.1^#^
Triglycerides (mg/dl)	158 ± 11.5	156 ± 10.9	143 ± 12.8	151 ± 20.2	122 ± 3.8	116 ± 3.4	155 ± 10.9	138 ± 9.4^∗##^
Cholesterol (mg/dl)	155 ± 8.2	163 ± 8.2	165 ± 6.1	152 ± 7.6^∗^	166 ± 4.9	158 ± 5.0^∗^	175 ± 7.4	163 ± 7.6^∗∗#^
HDL-C (mg/dl)	38.6 ± 2.1	40.6 ± 1.9	38.2 ± 1.8	40.3 ± 1.7	41.4 ± 1.5	43.4 ± 1.9	44.9 ± 2.8	48.1 ± 2.9^###*δδ*^
LDL-C (mg/dl)	114 ± 5.3	121 ± 5.2	127 ± 8.6	116 ± 9.2	133 ± 4.8	130 ± 5.2	138 ± 6.1	131 ± 6.6^##^
Creatinine (mg/dl)	1.0 ± 0.03	1.0 ± 0.04	1.0 ± 0.02	0.9 ± 0.02^∗^	1.1 ± 0.03	1.0 ± 0.03^∗∗^	1.1 ± 0.02	0.9 ± 0.02^∗∗^
Albumin (g/l)	3.9 ± 0.03	3.9 ± 0.04	4.0 ± 0.05	4.0 ± 0.03	4.1 ± 0.05	4.2 ± 0.07	4.1 ± 0.09	4.0 ± 0.06
SGOT (mg/dl)	28.9 ± 0.9	29.6 ± 1.3	26.5 ± 1.1	27.5 ± 1.4	30.5 ± 1.6	27.0 ± 2.0	27.2 ± 1.4	24.0 ± 0.9
SGPT (mg/dl)	24.8 ± 2.4	26.1 ± 2.6	29.2 ± 2.0	31.4 ± 2.4	28.4 ± 1.3	31.7 ± 1.5	21.2 ± 1.7	23.3 ± 2.0^###*δδδ*^
ALP (mg/dl)	250 ± 12.9	268 ± 13.2	263 ± 10.9	251 ± 9.3	205 ± 9.6	224 ± 10.5	218 ± 12.5	235 ± 10.1

The data are expressed as mean ± SEM. ^∗^Significantly vs. baseline, ^#^significantly vs. MitoQ follow-up, *^*δ*^*significantly vs. ET follow-up. ET: endurance training, FBS: fasting blood sugar, HDL-C: high-density lipoprotein, SGOT: serum glutamic-oxaloacetic transaminase, SGPT: serum glutamic-pyruvic transaminase, ALP: alkaline phosphatase.

## Data Availability

The data that support the findings of this study are available on request from the corresponding author. The data are not publicly available due to records which may contain information that could compromise patient confidentiality.
